# BELIEFS ABOUT AGING AND TRAUMA-INFORMED CARE WITHIN SKILLED NURSING FACILITIES

**DOI:** 10.1093/geroni/igae098.4130

**Published:** 2024-12-31

**Authors:** Melissa Meynadasy, Hannah Bashian, Jennifer Moye, Jennifer Sullivan, Whitney Mills, Jane Driver, Marcus Ruopp, Kelly O’Malley

**Affiliations:** VA Boston Healthcare System, West Roxbury, Massachusetts, United States; VA Boston Healthcare System, Boston, Massachusetts, United States; VA Boston Healthcare System, Boston, Massachusetts, United States; VA MC Providence, Providence, Rhode Island, United States; VA Providence Health Care System, VA Providence Health Care System, Rhode Island, United States; VA Boston Healthcare System, Brockton, Massachusetts, United States; VA Boston Healthcare System, Brockton, Massachusetts, United States; VA Boston Healthcare System, Boston, Massachusetts, United States

## Abstract

Pessimism about aging is ubiquitous and impacts emotional well-being and willingness to implement healthcare strategies with older adults. Individuals experiencing increased stress may be motivated to differentiate themselves from an older, more vulnerable group, which may further intensify pessimistic beliefs. This is particularly important to consider in skilled nursing facilities (SNFs) due to vulnerabilities residents experience. Healthcare environments/approaches can contribute to re-engagement with trauma symptoms, and pessimism about aging are related to trauma symptom endorsement. To explore how expectations about aging may impact staff perceptions of trauma-informed care (TIC), we assessed staff across disciplines (n = 78; nursing, rehabilitation, psychosocial, medical) in a sample of VA SNFs. Staff completed Expectations Regarding Aging (e.g., cognition, physical) and Attitudes Related to Trauma-Informed Care (e.g., beliefs about behavior) questionnaires. Higher total and subscale scores indicate positive expectations of aging and greater TIC endorsement. Expectations of aging differed significantly by discipline (F(3, 65) = 6.79, p <.001, ƞ2 =.24); nurses had lower expectations compared to other disciplines – and a significant difference compared to rehabilitation staff (p =.001; Bonferroni correction). Expectations about aging were not significantly related to overall perceptions of TIC (p =.09). However, staff expectations about cognitive functioning, were positively correlated with TIC attitudes about underlying causes of behavior (r =.25, p =.037), suggesting accurate knowledge about cognitive aging relates to more accurate understanding of resident behavior. This preliminary work suggests beliefs about aging may be related to some but not all aspects of TIC attitudes.

